# Characterization and Functional Analysis of *Chalcone Synthase* Genes in Highbush Blueberry (*Vaccinium corymbosum*)

**DOI:** 10.3390/ijms241813882

**Published:** 2023-09-09

**Authors:** Zening Zhang, Pengyan Qu, Siyi Hao, Ruide Li, Yongyan Zhang, Qi Zhao, Pengfei Wen, Chunzhen Cheng

**Affiliations:** College of Horticulture, Shanxi Agricultural University, Jinzhong 030801, China

**Keywords:** blueberry, chalcone synthase, gene expression, anthocyanin, transcription regulation

## Abstract

Chalcone synthase (CHS) is the first key enzyme-catalyzing plant flavonoid biosynthesis. Until now, however, the blueberry CHS gene family has not been systematically characterized and studied. In this study, we identified 22 *CHS* genes that could be further classified into four subfamilies from the highbush blueberry (*Vaccinium corymbosum*) genome. This classification was well supported by the high nucleotide and protein sequence similarities and similar gene structure and conserved motifs among VcCHS members from the same subfamily. Gene duplication analysis revealed that the expansion of the blueberry CHS gene family was mainly caused by segmental duplications. Promoter analysis revealed that the promoter regions of *VcCHSs* contained numerous *cis*-acting elements responsive to light, phytohormone and stress, along with binding sites for 36 different types of transcription factors. Gene expression analysis revealed that Subfamily I *VcCHSs* highly expressed in fruits at late ripening stages. Through transient overexpression, we found that three *VcCHSs* (*VcCHS13* from subfamily II; *VcCHS8* and *VcCHS21* from subfamily I) could significantly enhance the anthocyanin accumulation and up-regulate the expression of flavonoid biosynthetic structural genes in blueberry leaves and apple fruits. Notably, the promoting effect of the Subfamily I member *VcCHS21* was the best. The promoter of *VcCHS21* contains a G-box (CACGTG) and an E-box sequence, as well as a bHLH binding site. A yeast one hybridization (Y1H) assay revealed that three anthocyanin biosynthesis regulatory bHLHs (VcAN1, VcbHLH1-1 and VcbHLH1-2) could specifically bind to the G-box sequence (CACGTG) in the *VcCHS21* promoter, indicating that the expression of *VcCHS21* was regulated by bHLHs. Our study will be helpful for understanding the characteristics and functions of blueberry *CHSs.*

## 1. Introduction

Chalcone synthase (CHS) is a key enzyme catalyzing the first committed step of flavonoid biosynthesis. Since the first discovery of *CHS* in parsley (*Petroselinum hortense*) in 1983 [1], *CHSs* have been successfully isolated from many plant species, such as *Arabidopsis thaliana* [2], *Ipomoea purpurea* [3], *Gerbera hybrida* [4], *Chrysanthemum nankingense* [5] and *Zea mays* [6]. Their contributions to the flavonoid metabolism of many plants were widely proved [7,8]. For instance, a significant positive correlation between the expression of *CHS* genes and accumulations of anthocyanin has been reported in kiwifruit [9]; the overexpression of the *Freesia hybrid FhCHS1* gene in petunia altered the flower color from white to pink [10]; the virus-induced gene silencing (VIGS) of *Actinidia eriantha AeCHS* resulted in reduced petal anthocyanin accumulation and bleached red petals [11]; the silencing of the *CHS* gene of apple *(Malus × domestica*) resulted in the loss of pigmentation in fruit peel, flower and stem [12]; the silencing of the strawberry (*Fragaria × ananassai*) *FaCHS* gene resulted in the blocked accumulation of anthocyanins and the appearance of white areas in strawberries [13].

Evidence has revealed that many transcription factors (TFs) function in regulating flavonoids and anthocyanins biosynthesis by influencing the expression of *CHS* and some other structural genes. For example, *Lilium lancifolium* LlMYB3 could bind to the promoter of *LlCHS2* to improve its expression, and finally increased the accumulation of anthocyanins [14]. The apple (*M. domestica*) MdBBX22–MdHY5 interaction enhanced the binding ability of MdHY5 to the promoters of *MdMYB10* and *MdCHS*, thus promoting the anthocyanin biosynthesis [15]. *Glycine max* GmMYB176 could recognize and bind to the TAGT(T/A)(A/T) motif sequence within the *GmCHS8* promoter to promote the flavonoid biosynthesis in soybean [16]. *Solanum melongena* SmbHLH13 could activate the promoters of *SmCHS* and *SmF3H* and positively regulate their expression and flavonoid biosynthesis [17]. Both *Dendrobium officinale* DoMYB5 and DobHLH24 could directly bind to the promoters of *DoCHS* and *DoDFR* and regulate their expression. Their co-transformation could significantly upregulate the expression levels of *DoCHS* and *DoDFR* [18].

Blueberry (*Vaccinium corymbosum* L.) is a perennial shrub belonging to the genus Vaccinium of the family Ericaceae. Its fruits are rich with anthocyanins and it is one of the most ideal materials for anthocyanin metabolism research [19,20,21]. Plant *CHSs* usually had multi-gene family characteristics. However, reports on blueberry *CHSs* were mostly limited to single-gene cloning and quantitative real time PCR and/or comparative transcriptomic analysis-based expression analysis. For example, Lin et al. [22] found that the anthocyanin accumulation and expression of anthocyanin biosynthetic structural genes, including *CHS*, were high in the peel of blueberry fruits; through transcriptomic analysis of Northern Highbush (*V. corymbosum*) and Rabbiteye (*V. virgatum*) blueberry fruits, Günther et al. [23] reported that the interactions between flavonoid biosynthesis structural genes such as *CHS* and anthocyanin biosynthesis regulatory TFs contributed greatly to the anthocyanin biosynthesis; Han et al. [24] reported that the *CHS* gene expression was higher in ABA-treated blueberries than in untreated controls at late ripening stages, and their expression levels were significantly positively correlated with anthocyanin content. Up to now, however, systematic analysis of the blueberry CHS gene family has not been reported. In this study, we performed genome-wide identification of blueberry *CHS* genes, characterized their nucleotide and amino acid sequences and investigated their expression patterns in fruits at different ripening stages based on our previously obtained transcriptome data and quantitative real-time PCR (qRT-PCR) analysis. Meanwhile, blueberry leaf and apple fruit-based transient overexpression experiments were conducted for studying the functions of the three *VcCHS* genes (*VcCHS8*, *VcCHS13* and *VcCHS21*) that highly expressed in blueberry fruits at late ripening stages. In addition, a yeast one hybridization (Y1H) assay was applied to study the binding ability of anthocyanin biosynthesis regulatory blueberry bHLHs to the promoter of *VcCHS21*. Our study will be helpful for understanding the characteristics and functions of *CHS* genes in blueberry.

## 2. Results

### 2.1. Identification and Physiochemical Property Analysis of Blueberry CHSs

In total, 22 VcCHS proteins were identified from highbush blueberry. According to the chromosome location information of the coding genes, they were named VcCHS1~VcCHS22. Bioinformatic analysis results (Table 1) showed that the length in VcCHS proteins ranged from 383 aa (VcCHS17) to 557 aa (VcCHS5), with molecular weight ranging from 42.05 kDa (VcCHS17) to 61.13 kDa (VcCHS5); the isoelectric point ranged from 5.30 (VcCHS19) to 8.26 (VcCHS5). All 22 VcCHS proteins were identified to be hydrophilic proteins, and all blueberry CHSs except VcCHS5, VcCHS10, VcCHS11, VcCHS19 and VcCHS20 were predicted to be stable proteins. Subcellular localization prediction results revealed that all VcCHSs are cytoplasm-localized.

**Figure 1:**
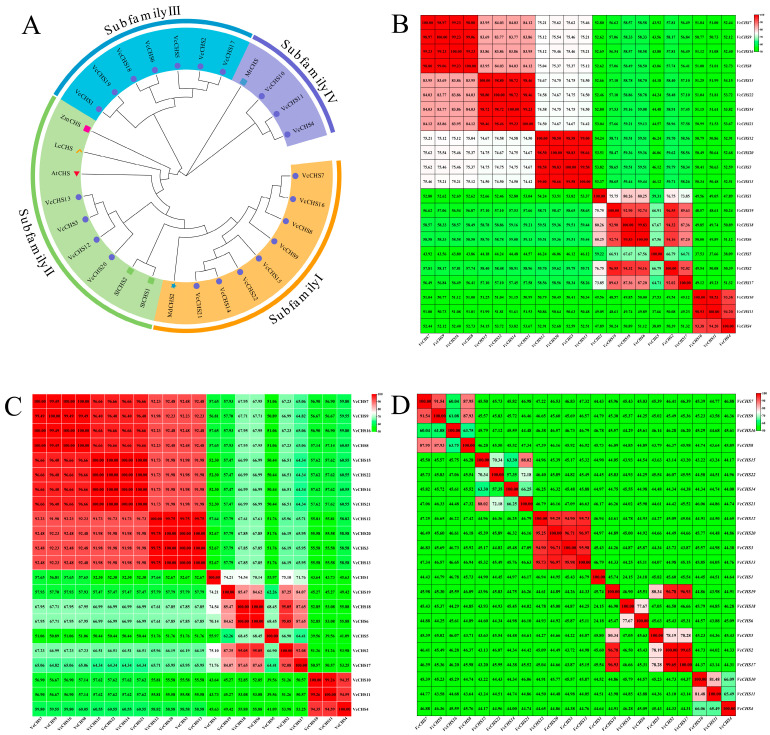
Phylogenetic (**A**) and sequence alignment (**B**–**D**) results of blueberry CHSs. (**A**): Phylogenic analysis results of CHSs from blueberry and some other plants. Vc: *V. corymbosum*; At: *A. thaliana*; Lc: *L. chinensis*; Zm: *Z. mays*; Mt: *M. truncatula*; Md: *M. domestica*; Sl: *S. lycopersicum*. (**B**): Nucleotide sequences similarities among *VcCHSs*; (**C**): Protein sequences similarities among VcCHSs; (**D**): Sequences similarities among *VcCHS* promoters. The redder the color, the higher the similarity, and the greener the color, the lower the similarity.

Phylogenetic analysis showed that VcCHSs could be divided into four subfamilies (Figure 1A). Subfamily I included eight VcCHSs that clustered with *M. domestica* MdCHS; Subfamily II contained four VcCHSs that were close to two *Solanum lycopersicum* SlCHSs, one *A. thaliana* AtCHS, one *Litchi chinensis* LcCHS and one *Z. mays* ZmCHS; Subfamily III contained seven VcCHSs; and Subfamily IV contained three VcCHSs that clustered together with *Medicago truncatula* MtCHS.

Sequence alignment results showed that the nucleotide similarities among 22 *VcCHSs* ranged from 37.53% to 99.83% (Figure 1B). The nucleotide similarities among Subfamily I members ranged from 83.69% to 99.23%. The nucleotide similarities among Subfamily II members were all very high, ranging from 98.50% to 99.58%. The nucleotide similarities among Subfamily III members ranged from 59.22% to 99.83%. The nucleotide similarities among the Subfamily IV members were also very high (93.38~99.53%).

The similarities of the 22 *VcCHSs* encoded proteins ranged from 39.56% to 100% (Figure 1C). The similarities among Subfamily I members ranged from 96.40% to 100% (the similarities among VcCHS16 and VcCHS7, VcCHS8 and VcCHS9, and among VcCHS14, VcCHS15, VcCHS21 and VcCHS22, were all 100%). The similarities among Subfamily II members were all higher than 99% (99.75~100%, with 100% similarity among VcCHS3, VcCHS13 and VcCHS20). The similarities of Subfamily III members ranged from 55.97% to 100% (VcCHS6 and VcCHS18), and the similarities of Subfamily IV members ranged from 94.35% to 99.26% (VcCHS10 and VcCHS11).

The 2000 bp sequences upstream from the start codons of *VcCHSs* were considered promoters. Promoter similarity analysis revealed that the similarities of the 22 *VcCHSs* promoters ranged from 24.15% to 99.65% (Figure 1D), among which, the similarities among promoters of Subfamily I, II, III and IV members was 60.04~91.54% (*VcCHS7* and *VcCHS9* promoters), 94.90~96.97% (*VcCHS13* and *VcCHS20* promoters), 24.15~99.65% (*VcCHS2* and *VcCHS17* promoters) and 65.49~81.48% (*VcCHS10* and *VcCHS11* promoters), respectively.

### 2.2. Gene Duplication and Synteny Analysis of Blueberry CHS Genes

**Figure 2:**
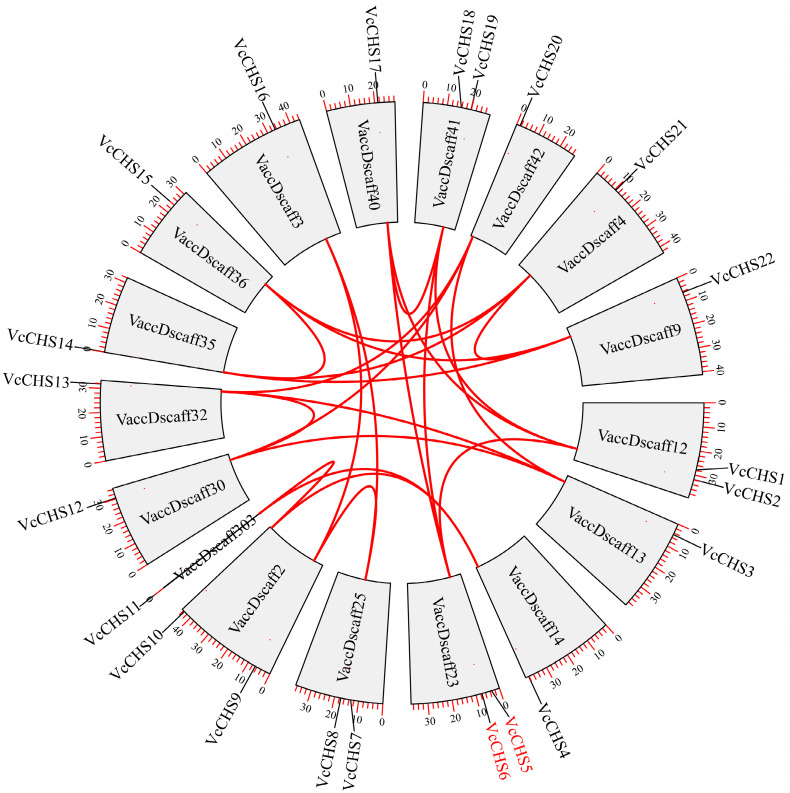
Chromosome localization and synteny analysis results of *VcCHSs*. The red lines represent segmental duplicated *VcCHS* gene pairs. The gene names in red are tandem duplicated gene pairs.

Gene duplication analysis revealed that there were 21 pairs of gene duplication events involving 18 (81.8%) *VcCHSs* (Table 2), including 20 pairs of segmental duplication genes and one pair of tandem duplication genes (*VcCHS5* and *VcCHS6*). Nine pairs of the segmental duplicated genes were from Subfamily I (*VcCH7* and *VcCHS16*; *VcCHS7* and *VcCHS9*; *VcCHS9* and *VcCHS16*; *VcCHS14* and *VcCHS15*; *VcCHS14* and *VcCHS21*; *VcCHS14* and *VcCHS22*; *VcCHS15* and *VcCHS21*; *VcCHS15* and *VcCHS22*; *VcCHS21* and *VcCHS22*), six pairs from Subfamily II (*VcCHS3* and *VcCHS12*; *VcCHS3* and *VcCHS13*; *VcCHS3* and *VcCHS20*; *VcCHS12* and *VcCHS13*; *VcCHS12* and *VcCHS20*; *VcCHS13* and *VcCHS20*), three pairs from Subfamily IV *(VcCHS4* and *VcCHS10*; *VcCHS4* and *VcCHS11*; *VcCHS10* and *VcCHS11*) and two pairs from Subfamily III (*VcCHS1* and *VcCHS5*, *VcCHS5* and *VcCHS18*). There were two gene duplications involving four *VcCHS* members (*VcCHS12*, *VcCHS13*, *VcCHS20* and *VcCHS3*; *VcCHS15*, *VcCHS21*, *VcCHS22* and *VcCHS14*). The Ka/Ks values of all duplicated gene pairs ranged from 0 to 0.5090. By calculating the divergence times of these duplicated *VcCHS* gene pairs, it was found that these duplication events occurred at 0.67 Mya to 7.93 Mya. Chromosome localization and synteny analysis showed that these 22 *VcCHSs* were localized to 17 scaffolds, and the tandem duplicated gene pair, *VcCHS5* and *VcCHS6*, was localized to VaccDscaff23 (Figure 2).

### 2.3. Conserved Motifs of VcCHSs and Gene Structures of Their Corresponding Genes

**Figure 3:**
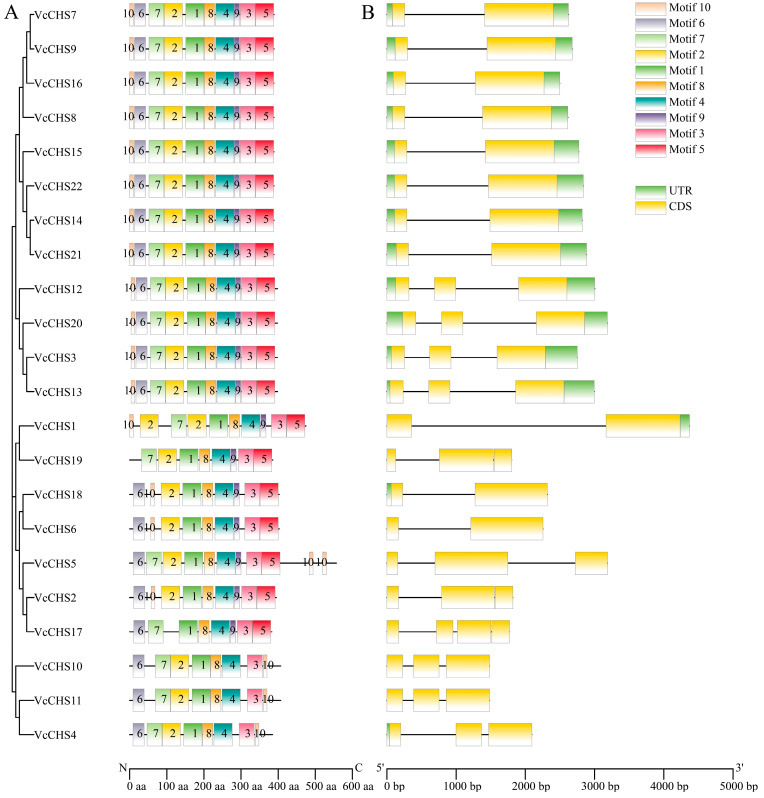
Conserved motif distributions in blueberry CHS proteins (**A**) and gene structures of their corresponding genes (**B**). UTR: untranslated region; CDS: coding sequence; aa: amino acid; bp: base pair; N: the N terminal of protein; C: the C terminal of protein.

By using MEME, we identified 10 motifs from blueberry CHSs, and Motif1, motif8, motif4 and motif3 were found in all VcCHSs (Figure 3A). However, VcCHS17 did not contain motif2 and motif10; VcCHS19 did not contain motif6 and motif10; VcCHS1 did not contain motif6; VcCHS2, VcCHS6 and VcCHS18 did not contain motif7; and Subfamily IV members did not contain motif5 and motif9. All the other VcCHSs contained 10 motifs arranged in the same order: motif10-6-7-2-1-8-4-9-3-5.

By analyzing the gene structures of blueberry *CHS* family genes, all *VcCHS* members had introns (Figure 3B). The Subfamily I members contained only one intron. Subfamily II and Subfamily IV members all contained two introns. The number of introns in Subfamily III members ranged from one to three. *VcCHS17* contained three introns; *VcCHS2*, *VcCHS5* and *VcCHS19* contained two introns; and *VcCHS1*, *VcCHS6* and *VcCHS18* contained only one intron.

### 2.4. Promoter Analysis Results of Blueberry CHS Genes

**Figure 4:**
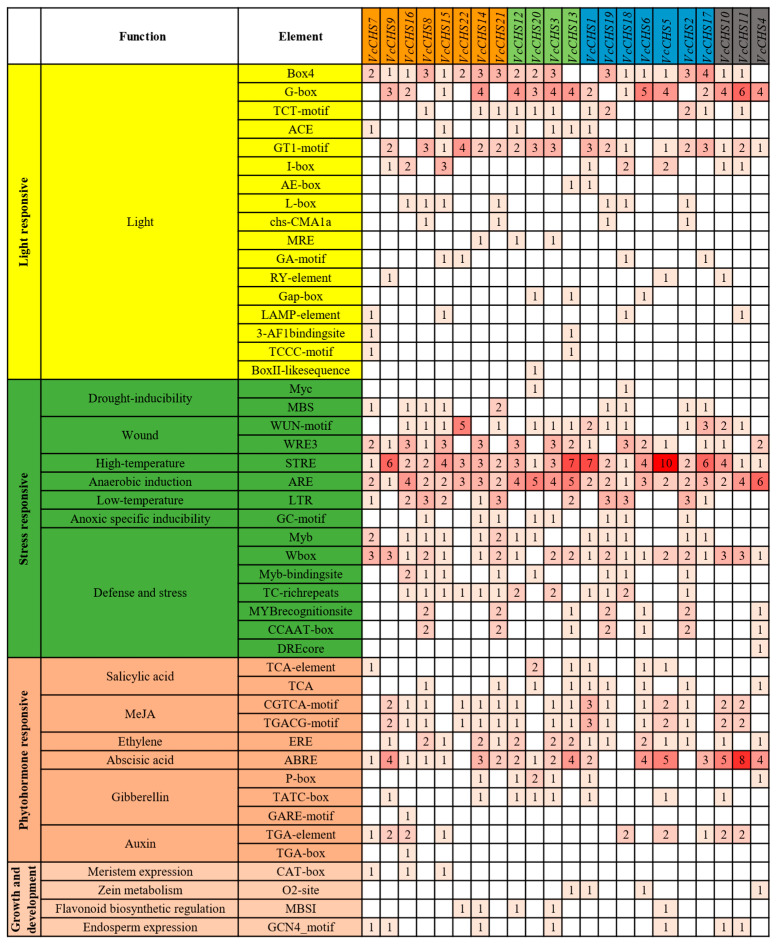
Predicted *cis*-acting elements in the promoters of *VcCHSs*.

Studies have found that *CHS* promoters contain many light-responsive elements [25]. Consistently, by analyzing the *cis*-acting elements in the *VcCHS* promoters, 17 kinds of light-responsive elements with a total number of 111 were identified (Figure 4). Notably, 19 (86.4%) *VcCHS* promoters contained the Box 4 element, 18 (81.8%) promoters contained the GT1-motif element and 17 members (77.3%) had the G-box element on their promoters. The CACGTG sequence is a bHLH binding site in the promoters of *CHS* genes [26]. Further sequence search revealed that this sequence could be found in promoters *VcCHS7*, *VcCHS8*, *VcCHS9*, *VcCHS10*, *VcCHS11* and *VcCHS15*, which were predicted to be of the G-box. Although the G-box element was not predicted on the *VcCHS21* promoter, there was a G-box (CACGTG) in its promoter, suggesting that bHLH could also bind to its promoter.

The *VcCHS* promoters also had many phytohormone-responsive elements. Notably, 18 (81.8%) *VcCHS* promoters had an abscisic acid (ABA)-responsive element. Moreover, there were 16 (72.7%), 15 (68.2%), 9 (40.9%), 9 (40.9%) and 8 (36.4%) *VcCHS* members containing MeJA-, ERE-, SA- auxin- and GA-responsive elements in their promoters, respectively.

The *VcCHSs* promoters also had many stress-responsive elements. All *VcCHSs* had high temperature and anoxic specific inducibility-related elements in their promoters. Additionally, 20 (90.9%), 16 (72.7%), 11 (50%), 9 (40.9%) and 8 (36.4%) members had defense and stress-related, wounding-related, low temperature-responsive, drought-inducibility-related and anoxic specific inducibility-related elements in their promoters, respectively. Additionally, we also identified some growth and development-related *cis*-acting elements in the promoters of *VcCHSs*, such as the meristem expression-related element, the zein metabolism regulation-related element and so on.

**Figure 5:**
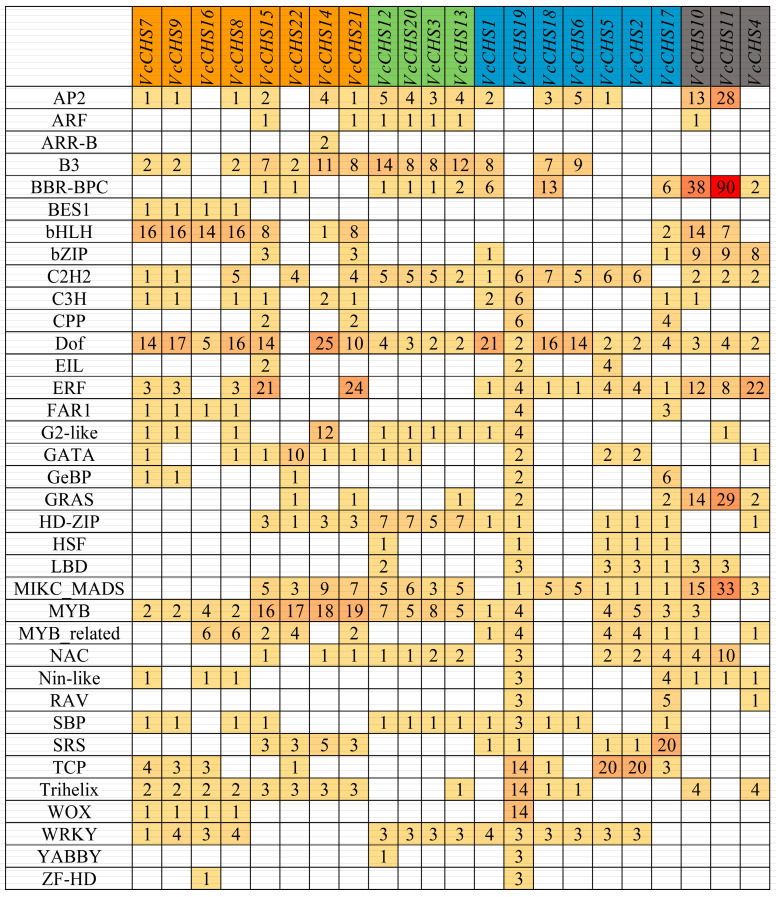
Predicted transcription factor binding sites in the promoters of *VcCHS* genes.

We further analyzed the transcription factor binding sites in the promoters of *VcCHS* genes (Figure 5). In total, we identified binding sites for 36 kinds of TFs. In promoters of all *VcCHSs*, the number of Dof binding sites was the largest (173), followed by MYB (122), and the binding site number of ARR-B was the smallest (only 2). Binding sites for twenty-eight kinds of TFs were identified in the *VcCHS19* promoter, while in the promoter of *VcCHS6*, binding sites for only nine types of TFs were identified. Except for *VcCHS22*, all the other 21 *VcCHSs* had Dof binding sites in their promoters, and only the *VcCHS14* promoter had an ARR-B binding site. Notably, MYB and Trihelix binding sites were identified in promoters of all Subfamily I members, and the bHLH binding site was found in promoters of all Subfamily I members except *VcCHS22*.

### 2.5. Expression Analysis of VcCHS Genes in Blueberry Fruits

**Figure 6:**
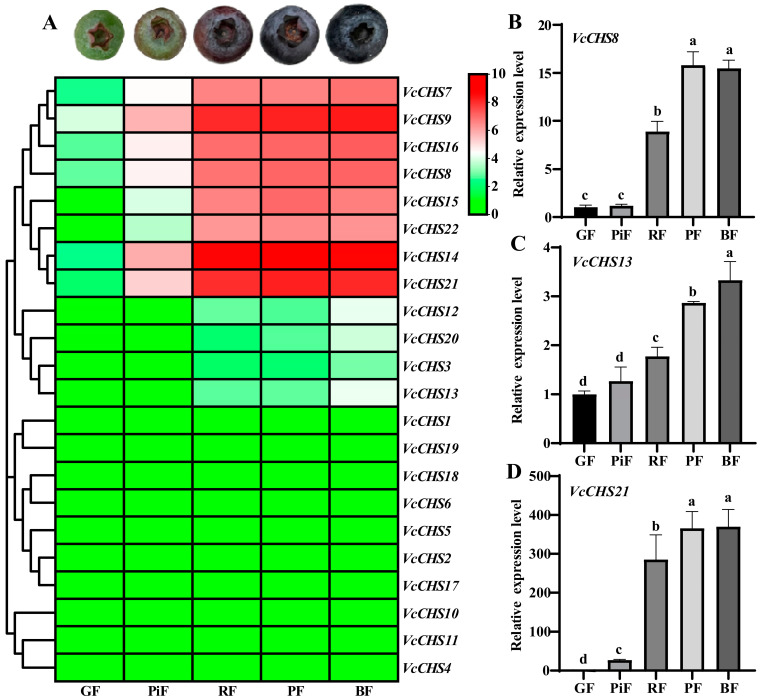
Gene expression analysis results of *VcCHSs*. (**A**): transcriptome data-based gene expression analysis of *VcCHSs.* For heatmap drawing, log_2_(FPKM + 1) values were used. The redder the color, the higher the gene’s expression; the greener the color, the lower the gene’s expression. (**B**–**D**): quantitative real time PCR analysis result for *VcCHS8*, *VcCHS13* and *VcCHS21*, respectively. GF: green fruit; PiF: pink fruit; RF: red fruit; PF: purple fruit; BF: blue fruit. Relative expression levels were calculated using GF as control (1). Different letters above columns represent significant difference at *p* < 0.05 level.

Transcriptome data-based gene expression analysis revealed that *VcCHS* members from Subfamily I had the highest expression levels in blueberry fruits, followed by Subfamily II members, while the Subfamily III and IV members expressed minimally, and some members (*VcCHS4*, *VcCHS10* and *VcCHS11*) had no expression in fruits at all ripening stages (Figure 6A). Noteworthily, Subfamily I and II members showed significantly higher expression levels in red, purple and blue fruits than in green and pink fruits, suggesting that they might play important roles in the biosynthesis of flavonoids and anthocyanin in blueberry fruits at late ripening stages.

Quantitative real time PCR (qRT-PCR) was further applied to validate the expression of three genes (*VcCHS8* and *VcCHS21* from Subfamily I, as well as *VcCHS13* from Subfamily II) that highly expressed in blueberry fruits at late ripening stages. Consistent with our transcriptome data, the expression levels of the three *VcCHSs* in fruits at late ripening stages (RF, PF and BF) were significantly higher than in GF and PiF. The expression of *VcCHS8* in RF, PF and BF was 8.75-, 15.52- and 15.20-fold of that of GF (Figure 6B), respectively. The expression of *VcCHS13* in RF, PF and BF was 1.77-, 2.87- and 3.33-fold of that of GF (Figure 6C), respectively. The expression of *VcCHS21* in PiF, RF, PF and BF was significantly higher than that of GF (Figure 6D), accounting for about 27.11-, 285.42-, 365.92- and 370.40-fold of GF, respectively.

### 2.6. Effects of Transient Overexpression of VcCHS8, VcCHS13 and VcCHS21 on Flavonoids and Anthocyanin Accumulations in Blueberry Leaves

**Figure 7:**
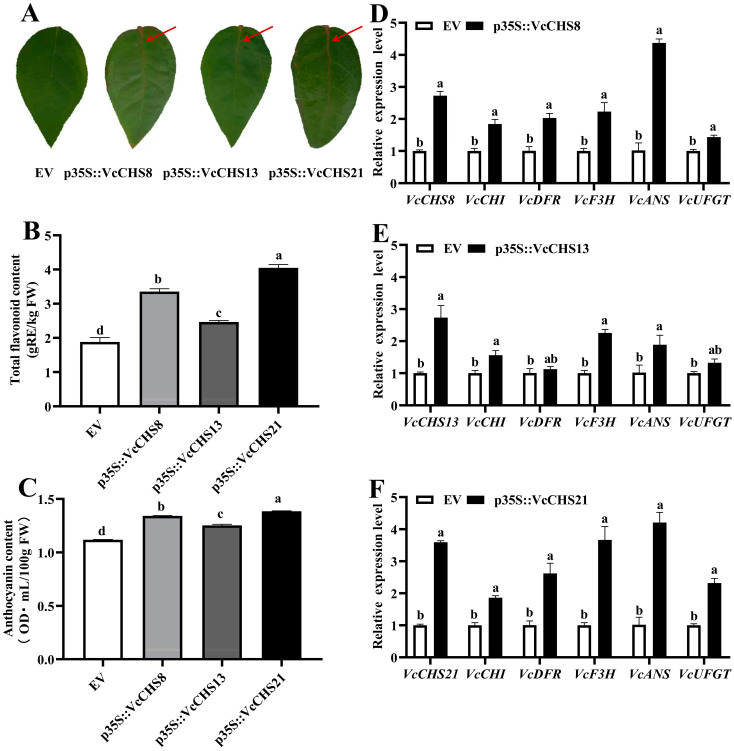
Effects of the transient overexpression of *VcCHS8*, *VcCHS13* and *VcCHS21* on the flavonoid biosynthesis in blueberry leaves. (**A**): blueberry leaves overexpressing EV, *VcCHS8*, *VcCHS13* and *VcCHS21*. Red arrows represent obvious pigmentation in veins. (**B,C**): influences of *VcCHS8, VcCHS13* and *VcCHS21* transient overexpression on flavonoid and anthocyanin contents in blueberry leaves. (**D**–**F**): influences of *VcCHS8, VcCHS13* and *VcCHS21* transient overexpression on the expression of flavonoid metabolism-related structural genes in blueberry leaves. FW: fresh weight; CHI: chalcone isomerase; FLS: flavonol synthase; F3H: flavanone 3-hydroxylase; DFR: dihydroflavonol 4-reductase; ANS: antho-cyanin synthase; UFGT: UDP-glucose: flavonoid 3-O-glucosyltransferase. Relative expression levels were calculated using EV as 1. Different letters above columns represent significant difference at *p* < 0.05 level.

To study the functions of *VcCHSs* in flavonoid and anthocyanin biosynthesis, blueberry leaf transient overexpression analysis of *VcCHS8*, *VcCHS13* and *VcCHS21* was performed. At five days post-vacuum inoculation, obvious pigmentation was observed in veins of blueberry leaves overexpressing *VcCHS8*, *VcCHS13* and *VcCHS21*, but not in empty vector (EV) transformed leaves (Figure 7A). Moreover, the vein of blueberry leaf overexpressing the Subfamily I member *VcCHS21* was the reddest. By determining the contents of flavonoids and anthocyanin in blueberry leaves (Figure 7B,C), we found that the flavonoid and anthocyanin content in blueberry leaves transiently overexpressing *VcCHS8*, *VcCHS13* and *VcCHS21* increased by 78.19%, 31.38% and 115.43%, and by 19.98%, 12.01% and 23.85%, respectively. It is worth noting that the promoting effects of Subfamily I members (*VcCHS8* and *VcCHS21*) were much better than Subfamily II member (*VcCHS13*), and the promoting effects of *VcCHS21* on flavonoids and anthocyanin accumulation were both significantly higher than *VcCHS8* and *VcCHS13* (*p* < 0.05).

QRT-PCR was further applied to investigate the influences of *VcCHS8*, *VcCHS13* and *VcCHS21* overexpression on the expression of flavonoid biosynthesis-related genes. Results showed that the expression levels of *VcCHS8*, *VcCHI, VcDFR, VcF3H, VcANS* and *VcUFGT* in blueberry leaves overexpressing *VcCHS8* were significantly higher than those of the EV (*p* < 0.05), accounting for approximately 1.56- 1.84-, 2.01-, 2.23-, 4.28- and 1.43-fold of the EV (*p* < 0.05), respectively (Figure 7D). The expression levels of *VcCHS13, VcCHI, VcF3H* and *VcANS* in blueberry leaves overexpressing *VcCHS13* were all significantly higher than those of the EV (*p* < 0.05), and the expression levels of *VcDFR* and *VcUFGT* were slightly higher than the EV, accounting for approximately 2.73-, 1.56-, 2.25-, 1.85-, 1.11- and 1.32-fold of the EV, respectively (Figure 7E). The expression levels of *VcCHS21, VcCHI, VcDFR, VcF3H, VcANS* and *VcUFGT* in blueberry leaves overexpressing *VcCHS21* were significantly higher than those of the EV (*p* < 0.05), accounting for approximately 3.59-, 1.86-, 2.59-, 3.67-, 4.13- and 2.32-fold of the EV, respectively (Figure 7F). It is obvious that the gene expression-promoting effect in blueberry leaves of *VcCHS21* overexpression was better than the other two *VcCHSs*.

### 2.7. Effects of Transient Overexpression of VcCHS8, VcCHS13 and VcCHS21 on Flavonoids and Anthocyanin Accumulations in Apple Fruits

**Figure 8:**
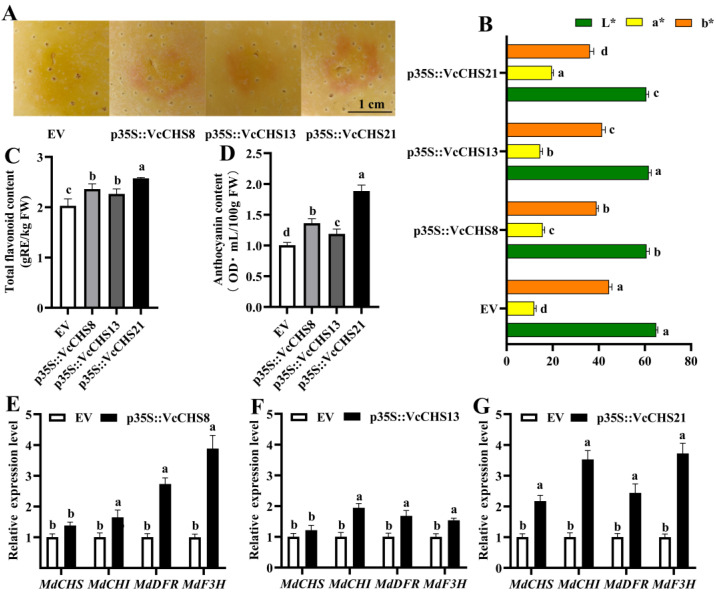
Effects of the transient overexpression of *VcCHS8*, *VcCHS13* and *VcCHS21* on flavonoid biosynthesis in apple fruits. (**A**): apple fruits overexpressing EV, *VcCHS8*, *VcCHS13* and *VcCHS21*. Bar = 1 cm. (**B**): color parameters of apple fruits overexpressing EV, *VcCHS8*, *VcCHS13* and *VcCHS21*. (**C**,**D**): influences of *VcCHS8*, *VcCHS13* and *VcCHS21* transient overexpression on flavonoid and anthocyanin contents in apple fruits. (**E**–**G**): influences of *VcCHS8, VcCHS13* and *VcCHS21* transient overexpression on the expression of flavonoid metabolism-related structural genes in apple fruits. Relative expression levels were calculated using EV as 1. Different letters above columns represent significant difference at *p* < 0.05 level.

Apple fruit transient transformation of VcCHS8, VcCHS13 and VcCHS21 was further conducted. No obvious pigmentation was observed in apple fruit transformed with EV (Figure 8A). However, pigmentations could be observed in apple fruits overexpressing these three VcCHSs, with apple fruit overexpressing VcCHS21 being the reddest. Fruit color index is an important index for fruit pigmentation, and the L*, a* and b* values of apple peels were firstly determined. Results showed that apple fruits overexpressing *VcCHS21* had the lowest L* and b* values but the highest a* value (Figure 8B). The transient overexpression of these three *VcCHSs* also increased the content of flavonoids and anthocyanin in apple fruits (Figure 8C,D). The flavonoid and anthocyanin content in apple fruits transiently overexpressing *VcCHS8*, *VcCHS13* and *VcCHS21* increased by 16.83%, 11.88% and 27.33%, and by 35.68%, 18.38% and 87.57%, respectively. It is worth noting that the content of flavonoids and anthocyanin in apple fruits transiently overexpressing *VcCHS21* were both significantly higher than fruits overexpressing *VcCHS8* or *VcCHS13* (*p* < 0.05).

The influence of *VcCHS8*, *VcCHS13* and *VcCHS21* overexpression on the expression of flavonoid and anthocyanin biosynthesis-related genes in apple fruits was further studied using qRT-PCR (Figure 8E–G). The results showed that the overexpression of *VcCHS8*, *VcCHS13* and *VcCHS21* all promoted the expression of *MdDFR*, *MdANS* and *MdUFGT* genes in the apple fruits. Interestingly, only the overexpression of *VcCHS21* significantly up-regulated the expression of *MdCHS* (*p* < 0.05).

### 2.8. Binding Ability of the Anthocyanin Biosynthesis Regulatory bHLH Transcription Factors to the VcCHS21 Promoter

**Figure 9:**
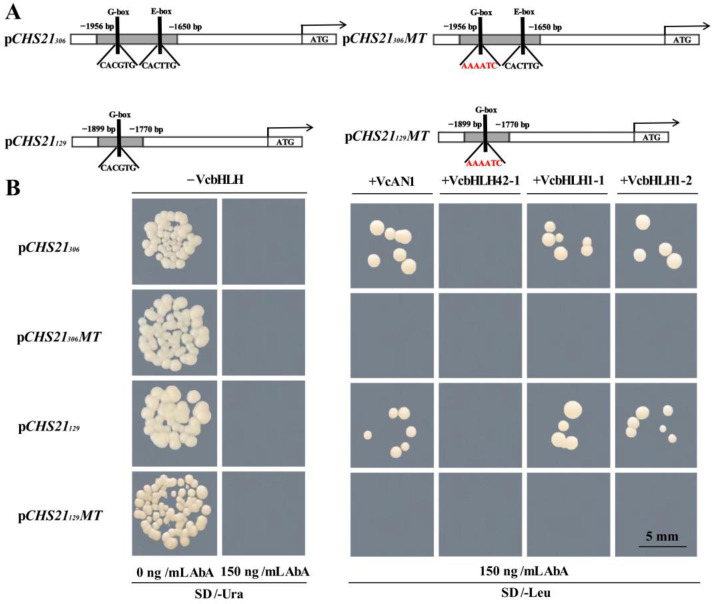
Y1H assays results for the binding abilities of anthocyanin regulatory bHLHs on the promoter of *VcCHS21*. (**A**): schematic diagrams for the cloned and mutated *VcCHS21* promoters. The *VcCHS21_306_* promoter contained both G-box and E-box sequences, while the *VcCHS21_129_* promoter carried only the G-box sequence. ‘CACGTG’ was mutated to ‘AAAATC’ in both p*CHS21_306_MT* and p*CHS21_129_MT*. (**B**): Y1H assay results. MT: mutant. Bar = 5 mm.

The bHLH transcription factors can activate the expression of flavonoid biosynthetic structural genes by binding to specific regions of their promoters, such as G-box (CACGTG) or E-box (CANNTG) [27]. According to the results of gene expression and transient overexpression analysis, it can be concluded that *VcCHS21* was a major *CHS* functioning in the flavonoids and anthocyanin biosynthesis of blueberry. The promoter of *VcCHS21* contains a G-box (CACGTG) sequence and bHLH binding site. Therefore, it was hypothesized that the expression of *VcCHS21* could be regulated by bHLHs. To verify this hypothesis, the binding activity of four previously identified blueberry anthocyanin biosynthesis regulatory bHLH transcription factors (VcAN1, VcbHLH42-1, VcbHLH1-1 and VcbHLH1-2) to the *VcCHS21* promoter was studied using yeast one hybridization (Y1H). The *VcCHS21_306_* promoter contained both G-box and E-box sequences, while the *VcCHS21_129_* promoter carried only the G-box sequence. The G-box sequence (CACGTG) was mutated to (AAAATC) in both p*CHS21_306_MT* and p*CHS21_129_MT* (Figure 9A). Y1H assay results showed that in the SD/–Ura medium containing 150 ng/mL AbA, the yeast strains separately transformed with p*CHS21_306_*, p*CHS21_129_*, p*CHS21_306_MT* and p*CHS21_129_MT*, and yeast strains co-transformed with p*CHS21_306_*/p*CHS21_129_* and VcbHLH42-1 could not grow; however, yeast strains co-transformed with p*CHS21_306_*/p*CHS21_129_* and VcAN1/VcbHLH1-1/VcbHLH1-2 grow well. These indicated that VcAN1, VcbHLH1-1 and VcbHLH1-2 could bind to the *VcCHS21* promoter. In addition, the yeast strains that co-transformed with p*CHS21_306_MT*/p*CHS21_129_MT* and VcAN1/VcbHLH42-1/VcbHLH1-1/VcbHLH1-2 could not grow on the SD/–Leu medium containing 150 ng/mL AbA, indicating that the G-box (CACGTG) was the specific binding sequence of VcAN1, VcbHLH1-1 and VcbHLH1-2 on the *VcCHS21* promoter (Figure 9B).

## 3. Discussion

### 3.1. The Expansion of Blueberry CHS Gene Family Is Mainly Caused by Segmental Duplications

The number of *CHS* members in different plant genomes varies greatly. For example, there is only one *CHS* gene in the *A. thaliana* genome [28]. However, there are six, eight, and fourteen *CHS* members in genomes of *I. purpurea* [3], *G. hybrida* [4] and *Z. mays* [6], respectively. In this study, we identified 22 *CHS* members from the highbush blueberry genome. Gene duplication, an important mechanism for the evolutionary expansion of gene families and acquiring novel genes and new functions, supports organisms adapting to diverse conditions [29]. The 16 *CHS* members in the *C. nankingense* genome included five pairs of segmental duplication genes and one pair of tandem duplication genes, indicating that segmental duplication contributed to the CHS gene family expansion in *C. nankingense* [5]. Consistent with this, by analyzing the gene duplication events occurred in blueberry CHS gene family, 20 pairs of segmental duplication involving 17 *CHS* members and one tandem duplicate (*VcCHS5* and *VcCHS6*) were found, indicating that segmental and tandem duplications, especially segmental duplication, contributed to the expansion of blueberry CHS gene family. In this present study, the Ka/Ks value of all duplicated *VcCHS* gene pairs was estimated to be ≤1.0, suggesting that the *VcCHS* family members survived the strong selection pressure of purification by elimination substitution and high selection pressure by natural selection during the evolutionary process [29,30]. Further, purifying selection can generate pseudogenes and/or genes with conserved functions [31,32]. Therefore, it can be inferred that the expansion of the CHS gene family contributed to the high flavonoid and anthocyanin characteristics of blueberry fruits.

### 3.2. The Subfamily I CHS Members Are Closely Related to Flavonoid and Anthocyanin Accumulation in Blueberry Fruits

Our study revealed that blueberry *CHS* genes can be divided into four subfamilies. The expression levels of Subfamily I and II members in blueberry fruits, especially Subfamily I members, were significantly higher than those from other subfamilies. Moreover, their expression levels in red, purple and blue fruits were significantly higher than those of green and pink fruits, suggesting that they played an important role in the biosynthesis of flavonoids and anthocyanin in blueberry fruits at late ripening stages.

Much research has revealed that the stable or transient overexpression of *CHS* genes could cause significant color changes of plants [33,34]. The blueberry fruit-based transient transformation system is unstable [35]. In this study, to verify the effects of the overexpression of *VcCHS8*, *VcCHS13* and *VcCHS21* genes, blueberry leaf and apple fruit transient transformation systems were applied. The results showed that all their transient overexpression promoted the accumulation of flavonoids and anthocyanin and significantly up-regulated the expression of flavonoid biosynthetic structural genes in both blueberry leaves and apple fruits. Moreover, only the transient overexpression of *VcCHS21* significantly up-regulated the expression of *MdCHS* in apple fruits. Notably, the promoting effects on accumulations of flavonoids and anthocyanin and the expression of flavonoid biosynthetic structural genes of Subfamily I members (*VcCHS8* and *VcCHS21*) were found to be better than the Subfamily II member (*VcCHS13*), with *VcCHS21* being the best. This indicated that the *VcCHS* members from Subfamily I play a more important role in the flavonoid and anthocyanin biosynthesis in blueberry fruits.

### 3.3. The Expression of the Blueberry CHSs Is Regulated by Transcription Factors

The biosynthesis of flavonoids and anthocyanin and the expression of their corresponding structural genes, including *CHS*, can be influenced by many internal or external stimuli [36,37,38,39,40,41]. In this study, we found 111 light-responsive elements in the promoters of 22 *VcCHSs*, indicating that their expression can be significantly influenced by light. Moreover, we also identified many phytohormone-responsive, defense and stress-related, wounding-related, low temperature-responsive and drought-inducibility-related elements in their promoters, suggesting that the expression of *VcCHSs* can be influenced greatly by many phytohormones and environmental factors.

TFs play an important role in regulating the biosynthesis of flavonoids and anthocyanin [42]. The expression of anthocyanin biosynthesis structural genes is usually regulated by the MYB-bHLH-WD40 (MBW) complex [43]. The regulatory functions of this complex can be achieved by binding to promoters of anthocyanin biosynthesis-related structural genes and regulating their expression [44]. In *L. lancifolium* [14] and *S. melongena* [45], MYB can bind to the MYB binding site in the promoter region of *CHS* to promote anthocyanin accumulation. GmMYB176 can specifically bind to the TAGT(T/A)(A/T) sequence on the *GmCHS8* promoter to promote flavonoid biosynthesis in soybean [16]. In this study, we identified binding sites for 36 transcription factors from the *VcCHS* promoters. It is worth noting that MYB binding sites exist on all Subfamily I members promoters, and bHLH binding sites exist on all Subfamily I members promoters except *VcCHS22*. Therefore, it can be inferred that the expression of Subfamily I *VcCHSs* may be regulated by MYB and bHLH.

bHLH can bind to the G/E-box in the promoter sequence of target genes [27]. In *Salvia miltiorrhiza*, SmbHLH60 could specifically bind to the G-box (CACGTG) in the *SmDFR* promoter and suppress its expression to reduce anthocyanin biosynthesis [26]. Sequence analysis revealed that there was both G-box (CACGTG) and E-box in the *VcCHS21* promoter. Y1H results revealed that three blueberry anthocyanin regulatory VcbHLHs (VcAN1, VcbHLH1-1-1 and VcbHLH1-2) could specifically bind to the G-box (CACGTG) on the *VcCHS21* promoter. This supports well the regulation of TFs on the expression of *VcCHSs*.

## 4. Materials and Methods

### 4.1. Identification of the Blueberry CHS Genes

The blueberry genome data were downloaded from the GENOMEDATABASE FOR VACCINIUM (GDV, https://www.vaccinium.org/, accessed on 8 March 2023) [46], and the protein sequence of *A. thaliana* CHS was downloaded from TAIR (https://www.arabidopsis.org/, accessed on 8 March 2023) and used to query BLASTP against the blueberry protein database under criterion of e-value ≤ 1 × 10^−5^. Meanwhile, the Hidden Markov Model files of CHS domains PF00195 (Clal_sti_synt_C) and PF02797 (Clal_sti_synt_N) downloaded from the Pfam database (http://pfam.xfam.org/, accessed on 10 March 2023) were used to identify CHS proteins from blueberry protein files by using HMMER 3.0 software embedded in TBtools (e-value ≤ 1 × 10^−5^) [47]. Candidate CHSs obtained through these two methods were further subjected to conserved domain confirmation using CDD (https://www.ncbi.nlm.nih.gov/Structure/cdd/wrpsb.cgi/, accessed on 10 March 2023), and only proteins containing both Clal_sti_synt_C and Clal_sti_synt_N domains were remained. One exception is that VaccDscaff22-snap-gene-48.42 was also removed because its protein length is more than 1300 aa.

### 4.2. Bioinformatic Analysis of Blueberry CHSs and Their Encoded Proteins

ExPASy-ProtParam tool (https://web.expasy.org/protparam/, accessed on 10 March 2023) was used to predict the amino acid number, protein molecular weight, isoelectric point, grand average of hydropathicity and instability index of blueberry CHS proteins. For the subcellular localization prediction of VcCHSs, WoLF PSORT (https://wolfpsort.hgc.jp/, accessed on 10 March 2023) was used [48].

### 4.3. Phylogenetic Analysis of Blueberry CHS Proteins

MEGA11 software (Molecular Evolutionary Genetics Analysis Version 11.0) was applied for the multiple sequence alignments of CHS proteins from *V. corymbosum*, *A. thaliana*, *Z. mays*, *Litchi chinensis*, *Medicago truncatula*, *Malus domestica* and *Solanum lycopersicum* and for phylogenetic tree construction by using the Neighbor Joining (NJ) method with default parameters (bootstrap = 1000). EvolView (https://www.evolgenius.info/evolview/#/, accessed on 10 March 2023) was used for figure drawing [49].

### 4.4. Synteny Analysis of Blueberry CHS Genes

Synteny analysis of *VcCHS* genes was performed using MCscanX (Multiple Collinearity Scan Toolkit X version). The ‘Circos’ of the TBtools v2.0 software was employed to display the collinear distribution of *VcCHSs* [50]. TBtools was used to analyzed the duplication events of *VcCHSs* [31], and the divergence time of duplicated gene pairs was calculated using the formula: T = Ks/2λ × 10^−6^ Mya (λ = 1.3 × 10^−8^) [46].

### 4.5. Gene Structure Analysis of VcCHSs and Conserved Motif analysis of Their Encoded Proteins

Gene structures of *VcCHSs* and conserved motifs in their encoded proteins were analyzed using GSDS (http://gsds.cbi.pku.edu.cn/, accessed on 10 March 2023) and MEME (https://meme-suite.org/meme/, accessed on 10 March 2023), respectively. For figure drawing, TBtools software was used [5].

### 4.6. Promoter Analysis

TBtools was used to extract the 2000 bp sequences upstream from the start codons (ATG) of *VcCHSs* and used as promoter sequences. The cis-acting elements and transcription factor binding sites in promoters of *VcCHSs* were analyzed using PlantCARE (http://bioinformatics.psb.ugent.be/webtools/plantcare/html/, accessed on 10 March 2023) and PlantTFDB (http://planttfdb.cbi.pku.edu.cn/, accessed on 10 March 2023) (*p*-value ≤ l × 10^−5^), respectively.

### 4.7. Gene Expression Analysis of VcCHSs

The FPKM (Fragments Per Kilobases per Million reads) values of *VcCHSs* were extracted from our transcriptome data, transformed into log_2_(FPKM + 1) and used for expression heatmap analysis using ‘HeatMap’ of TBtools [47]. In addition, for the expression validation of three highly expressed *VcCHS* genes (*VcCHS8*, *VcCHS13* and *VcCHS21*) in blueberry fruits at five different ripening stages (green, pink, red, purple and blue fruit stages), quantitative real-time PCR (qRT-PCR) was also performed. Primers used for qRT-PCR analysis are listed in Table S1.

### 4.8. Gene Cloning and Vector Construction

By using gene-specific primers (Table S1), *VcCHS8*, *VcCHS13* and *VcCHS21* genes were amplified using cDNA of ‘F3L’ blueberry as template. The amplified products were detected by 1.2% agarose gel electrophoresis, gel purified, ligated into the pMD18-T vectors, and transformed into component *E. coli* DH5α. Positive bacteria were sent to Shanghai Bioengineering Company (Shanghai, China) for sequencing confirmation. Gene-specific primer pairs with *Bam*HI digestion site sequences (GGATCC) in the forward primers and *Spe*I digestion site sequences (ACTAGT) in the reverse primers were designed and used for amplifying the sequences used for vector construction (Table S1). Amplified PCR products were individually ligated into *Bam*HI and *Spe*I double digested pBI121 vector using the Ready-to-use Seamless Cloning Kit (Sangon Biotech, Shanghai, China), and then transformed into *Agrobacterium tumefaciens* GV3101.

### 4.9. Transient Overexpression Analysis of VcCHS8, VcCHS13 and VcCHS21 in Blueberry Leaves and Apple Fruits

*A. tumefaciens* solution carrying target gene was transferred to 50 mL of LB liquid medium (containing 50 ng/mL Kan and 25 ng/mL Rif), cultured to OD_600_ = 0.8, concentrated at 5000 rpm for 5 min, resuspended in a suspension containing 200 μM AS,10 mM MgCl_2_ and 10 mM MES, adjusted to OD_600_ to 0.2~0.5 and incubated at room temperature for 1–2 h for further use.

Healthy and uniform blueberry leaves without mechanical injuries were submerged in *A. tumefaciens* inoculation solution, vacuumed for 20 min, cultured in the dark for 1 d and moved to normal light condition for five days. Then, total flavonoid and anthocyanin content in blueberry leaves was determined according to Fu et al. [51] and Zhang et al. [20], respectively.

Bagged ‘Gala’ apple fruits were also used for the transient overexpression analysis of *VcCHSs*. *A. tumefaciens* inoculation solution was injected into the central axis epidermis of apple fruit with a 1 mL syringe. The same fruit was injected with pBI121, pBI121-*VcCHS8*, pBI121-*VcCHS13* and pBI121-*VcCHS21* [52], cultured in the dark for 2 d and moved to normal light condition for five days. At 5 d post-treatment, color parameters (L*, a* and b* values) of fruit pericarps were measured using a CR8 Portable Colorimeter (Shenzhen Threenh Technology Co., Ltd., Shenzhen, China). Then, the total flavonoid and anthocyanin content in fruit peels was determined.

Total RNA was isolated from blueberry leaves and apple fruit peels using Trizol (Invitrogen, Waltham, CA, USA). PrimeScript RT Master Mix (Perfect Real Time) kit (Takara, Dalian, China) was used for biosynthesizing the complementary DNA (cDNA) used for quantitative real-time PCR. The expression of flavonoid biosynthesis-related genes (*VcCHS*, *VcCHI*, *VcDFR*, *VcF3H*, *VcANS* and *VcUFGT* for blueberry, *MdCHS, MdDFR*, *MdANS* and *MdUFGT* for apple [53]) was investigated on an ABI 7500 real-time PCR system. The reaction procedure was set as follows: pre-denaturation at 95 °C for 30 s; 45 cycles of denaturation at 95 °C for 30 s, annealing at 60 °C for 30 s and extension at 72 °C for 30 s. Three biological replications were made for each gene. *GAPDH* [54] and *Actin* [55] were used as internal reference genes for blueberry and apple gene expression analysis, respectively. The relative expression levels of these genes in different samples were calculated using the 2^−ΔΔCT^ method. Primers used for qRT-PCR analysis are listed in Table S1.

### 4.10. Yeast One-Hybrid (Y1H) Assay

Primers for amplifying the *VcCHS21* promoter (Table S1), the 306 bp promoter sequence (*CHS21_306_*) containing G-box/E-box and the 129 bp promoter sequence (*CHS21_129_*) containing only G-box were used to amplify target promoter sequences using gDNA of ‘F3L’ blueberry as template. By using the Ready-to-use Seamless Cloning Kit, amplified promoter sequences were ligated to *Sma*I and *Xho*I double-digested pAbAi vectors. Recombinant p*CHS21_306_* and p*CHS21_129_* plasmids were obtained and transformed into component *E. coli* DH5α. Meanwhile, synthetic promoter sequences with a mutant G-box sequence (CACGTG→AAAATC) were also ligated to the pAbAi vectors to obtain p*CHS21_306_MT* and p*CHS21_129_MT* vectors. Then, linearized p*CHS21_306_*, p*CHS21_129_*, p*CHS21_306_MT* and p*CHS21_129_MT* vectors (digested using *Bsp*T104I) were transformed into yeast strain Y1HGold and subjected to AbA concentration screening and autoactivation validation. *VcAN1* (VaccDscaff11-processed-gene-379.7), *VcbHLH42-1* (VaccDscaff24-augustus-gene-24.28), *VcbHLH1-1* (VaccDscaff28-augustus-gene-45.27) and *VcbHLH1-2* (VaccDscaff44-augustus-gene-0.19) hawere identified to be anthocyanin biosynthesis regulatory bHLHs in blueberry [21]. In this study, we cloned the four VcbHLH genes and introduced them to pGADT7 vector using Ready-to-use Seamless Cloning Kit (Sangon Biotech, Shanghai, China) to obtain recombinant pGADT7-VcAN1, pGADT7-VcbHLH42-1, pGADT7-VcbHLH1-1 and pGADT7-VcbHLH1-2 vectors. Y1H assay was performed to study the binding ability of these four VcbHLHs to the *VcCHS21* promoter by transforming pGADT7-VcAN1, pGADT7-VcbHLH42-1, pGADT7-bVcHLH1-1 and pGADT7-VcbHLH1-2 vectors into the yeast strain Y1HGold carrying p*CHS21_306_*_,_ p*CHS21_129_*, p*CHS21_306_MT* and p*CHS21_129_MT* [14], respectively.

## 5. Conclusions

In this study, the blueberry CHS gene family was systematically identified and analyzed. A total of 22 CHS members that can be further classified into four subfamilies were identified from highbush blueberry. Gene expression analysis revealed that the Subfamily I members are highly expressed in blueberry fruits at late ripening stages. The transient overexpression of *VcCHS8*, *VcCHS13* and *VcCHS21* could increase the accumulation of flavonoid anthocyanins in blueberry leaves and apple fruits, and the overexpression of the Subfamily I member *VcCHS21* showed the best promoting effect. Additionally, anthocyanin biosynthesis regulatory bHLHs (VcAN1, VcbHLH1-1-1 and VcbHLH1-2) could specifically bind to the G-box (CACGTG) sequence on the *VcCHS21* promoter, suggesting that these bHLHs could regulate blueberry flavonoids and anthocyanin biosynthesis by regulating the expression of *CHS*.

## Figures and Tables

**Table 1:** Physiochemical properties of blueberry CHS members. AA: amino acid; pI: isoelectric point; GRAVY: grand average of hydropathicity.

Gene Name	Gene ID	Number of AA	Molecular Weight/Da	pI	Instability Index	Aliphatic Index	GRAVY	Subcellular Localization
*VcCHS1*	VaccDscaff12-processed-gene-318.4	475	52,294.14	5.40	39.3	95.22	−0.132	Cytoplasm
*VcCHS2*	VaccDscaff12-snap-gene-319.33	395	43,328.65	5.55	38.54	96.53	−0.129	Cytoplasm
*VcCHS3*	VaccDscaff13-augustus-gene-46.32	398	43,199.79	5.92	38.68	90.63	−0.084	Cytoplasm
*VcCHS4*	VaccDscaff14-processed-gene-382.0	385	42,193.76	6.19	39.09	93.27	−0.143	Cytoplasm
*VcCHS5*	VaccDscaff23-processed-gene-74.2	557	61,129.82	8.26	47.69	87.34	−0.253	Cytoplasm
*VcCHS6*	VaccDscaff23-snap-gene-74.34	403	44,634.05	5.44	36.25	93.13	−0.163	Cytoplasm
*VcCHS7*	VaccDscaff25-augustus-gene-173.26	389	42,577.31	6.04	37.78	90.49	−0.082	Cytoplasm
*VcCHS8*	VaccDscaff25-augustus-gene-174.22	389	42,603.35	5.97	37.3	90.75	−0.078	Cytoplasm
*VcCHS9*	VaccDscaff2-augustus-gene-67.14	389	42,505.25	6.18	37.33	90.75	−0.068	Cytoplasm
*VcCHS10*	VaccDscaff2-processed-gene-435.12	407	44,652.54	5.97	44.56	93.02	−0.109	Cytoplasm
*VcCHS11*	VaccDscaff303-processed-gene-0.17	407	44,676.58	5.97	41.41	91.11	−0.116	Cytoplasm
*VcCHS12*	VaccDscaff30-augustus-gene-303.29	398	43,215.86	5.92	38.12	90.63	−0.076	Cytoplasm
*VcCHS13*	VaccDscaff32-augustus-gene-318.21	398	43,199.79	5.92	38.68	90.63	−0.084	Cytoplasm
*VcCHS14*	VaccDscaff35-augustus-gene-1.21	389	42,538.25	6.04	35.09	92.49	−0.045	Cytoplasm
*VcCHS15*	VaccDscaff36-augustus-gene-240.30	389	42,538.25	6.04	35.09	92.49	−0.045	Cytoplasm
*VcCHS16*	VaccDscaff3-augustus-gene-334.17	389	42,591.34	6.04	37.78	90.75	−0.083	Cytoplasm
*VcCHS17*	VaccDscaff40-snap-gene-213.40	383	42,048.07	5.56	38.31	92.95	−0.197	Cytoplasm
*VcCHS18*	VaccDscaff41-snap-gene-198.34	403	44,647.09	5.53	36.46	93.37	−0.162	Cytoplasm
*VcCHS19*	VaccDscaff41-snap-gene-198.39	386	42,379.59	5.3	41.41	104.33	−0.097	Cytoplasm
*VcCHS20*	VaccDscaff42-augustus-gene-14.30	398	43,227.81	5.92	40.12	90.63	−0.086	Cytoplasm
*VcCHS21*	VaccDscaff4-processed-gene-104.3	389	42,538.25	6.04	35.09	92.49	−0.045	Cytoplasm
*VcCHS22*	VaccDscaff9-processed-gene-64.0	389	42,538.25	6.04	35.09	92.49	−0.045	Cytoplasm

**Table 2:** Gene duplication analysis results of blueberry *CHS* genes. Mya: million years ago. For abbreviation, ‘VaccDscaff’ is removed from all gene IDs in this table.

Gene ID	Gene Name	Gene ID	Gene Name	Ka	Ks	Ka_Ks	Duplication Date/Mya	Duplication Type
23-processed-gene-74.2	*VcCHS5*	23-snap-gene-74.34	*VcCHS6*	0.0413	0.1090	0.3788	4.19	Tandem duplication
12-processed-gene-318.4	*VcCHS1*	23-processed-gene-74.2	*VcCHS5*	0.1050	0.2063	0.5090	7.93	Segmental duplication
2-processed-gene-435.12	*VcCHS10*	303-processed-gene-0.17	*VcCHS11*	0.0065	0.0423	0.1534	1.63	Segmental duplication
30-augustus-gene-303.29	*VcCHS12*	32-augustus-gene-318.21	*VcCHS13*	0.0011	0.0388	0.0286	1.49	Segmental duplication
30-augustus-gene-303.29	*VcCHS12*	42-augustus-gene-14.30	*VcCHS20*	0.0022	0.0570	0.0390	2.19	Segmental duplication
32-augustus-gene-318.21	*VcCHS13*	42-augustus-gene-14.30	*VcCHS20*	0.0011	0.0533	0.0208	2.05	Segmental duplication
35-augustus-gene-1.21	*VcCHS14*	36-augustus-gene-240.30	*VcCHS15*	0	0.0569	0	2.19	Segmental duplication
35-augustus-gene-1.21	*VcCHS14*	4-processed-gene-104.3	*VcCHS21*	0	0.0336	0	1.29	Segmental duplication
35-augustus-gene-1.21	*VcCHS14*	9-processed-gene-64.0	*VcCHS22*	0	0.0569	0	2.19	Segmental duplication
36-augustus-gene-240.30	*VcCHS15*	4-processed-gene-104.3	*VcCHS21*	0	0.0688	0	2.65	Segmental duplication
36-augustus-gene-240.30	*VcCHS15*	9-processed-gene-64.0	*VcCHS22*	0	0.0530	0	2.04	Segmental duplication
4-processed-gene-104.3	*VcCHS21*	9-processed-gene-64.0	*VcCHS22*	0	0.0688	0	2.65	Segmental duplication
13-augustus-gene-46.32	*VcCHS3*	30-augustus-gene-303.29	*VcCHS12*	0.0011	0.0571	0.0194	2.19	Segmental duplication
13-augustus-gene-46.32	*VcCHS3*	32-augustus-gene-318.21	*VcCHS13*	0	0.0174	0	0.67	Segmental duplication
13-augustus-gene-46.32	*VcCHS3*	42-augustus-gene-14.30	*VcCHS20*	0.0011	0.0460	0.0241	1.77	Segmental duplication
14-processed-gene-382.0	*VcCHS4*	2-processed-gene-435.12	*VcCHS10*	0.0046	0.0449	0.1017	1.73	Segmental duplication
14-processed-gene-382.0	*VcCHS4*	303-processed-gene-0.17	*VcCHS11*	0	0.0221	0	0.85	Segmental duplication
23-processed-gene-74.2	*VcCHS5*	41-snap-gene-198.34	*VcCHS18*	0.0390	0.1091	0.3575	4.20	Segmental duplication
25-augustus-gene-173.26	*VcCHS7*	3-augustus-gene-334.17	*VcCHS16*	0.0011	0.0298	0.0376	1.15	Segmental duplication
2-augustus-gene-67.14	*VcCHS9*	25-augustus-gene-173.26	*VcCHS7*	0.0034	0.0336	0.1003	1.29	Segmental duplication
2-augustus-gene-67.14	*VcCHS9*	3-augustus-gene-334.17	*VcCHS16*	0.0045	0.0185	0.2434	0.71	Segmental duplication

## Data Availability

All data are available in this article and its Supplementary Materials.

## Supplementary Materials

The supporting information can be downloaded at: https://www.mdpi.com/article/10.3390/ijms241813882/s1.
