# Supplementary material for: Characterization and Functional Analysis of Chalcone Synthase Genes in Highbush Blueberry (Vaccinium corymbosum)
Source: Int J Mol Sci. 2023 Sep 9;24(18):13882. doi: 10.3390/ijms241813882 (PMC10530253; doi:10.3390/ijms241813882)
Supplement: Supplementary file 1 [file ijms-24-13882-s001.zip › ijms-2595200-supplementary.pdf]

Table S1 Information for the primers used in this study.

Primer Name	Forward Primer	Reverse Primer	Applications
VcCHS8	ATGGTCACCGTCGAGGAA	TCAAGTGCACAAGCTGTGG	
VcCHS8-OE	ACGGGGGACTCTAGAGGATCCATGGTCACCGTCGAGGAA	GCTCACCATCGCTGCACTAGTTCAAGTGCACAAGCTGTGG	
VcCHS13	ATGGCTCCTACAGGTGTGT	TCAGTGAGTCACACCGC	
VcCHS13-OE	ACGGGGGACTCTAGAGGATCCATGGCTCCTACAGGTGTGT	GCTCACCATCGCTGCACTAGTTCAGTGAGTCACACCGC	
VcCHS21	ATGGTGACCGTCGAGGAA	CTAAGTGCACAGACTATGGAGC	
VcCHS21-OE	ACGGGGGACTCTAGAGGATCCATGGTGACCGTCGAGGAA	GCTCACCATCGCTGCACTAGTCTAAGTGCACAGACTATGGAGC	
VcCHS21$_{306}$	TATTTTTGCTAGTAATAATATGAGT	AGAAATAAAAATAAAAAAATTTAAATTTGG	
VcCHS21$_{306}$-Ai	AATTCGAGCTCGGTACCCGGGTATTTTTGCTAGTAATAATATGAGT	ATACAGAGCACATGCCTCGAGAGAAATAAAAATAAAAAAATTTAAATTTGG	
VcCHS21$_{129}$	AGTTGAAACTCAGCTTG	TTTGACATCTCTCTTTAATC	
VcCHS21$_{129}$-Ai	AATTCGAGCTCGGTACCCGGGAGTTGAAACTCAGCTTG	ATACAGAGCACATGCCTCGAGTTTGACATCTCTCTTTAATC	Vector construction
VcAN1	GCAACCCTCTCTCTTTCACT	CCATTTCATTACCGCAAGC	
VcAN1-AD	GCCATGGAGGCCAGTGAATTCGCAACCCTCTCTCTTTCACT	CAGCTCGAGCTCGATGGATCCCCATTTCATTACCGCAAGC	
VcbHLH42-1	GCAACCCTCTCTCTTTCACT	CCATTTCATTACCGCAAGC	
VcbHLH42-1-AD	GCCATGGAGGCCAGTGAATTCGCAACCCTCTCTCTTTCACT	CAGCTCGAGCTCGATGGATCCCCATTTCATTACCGCAAGC	
VcbHLH1-1	GCACAGAATCAATGGCTTC	CGTGTTTGTTGGTTTGGC	
VcbHLH1-1-AD	GCCATGGAGGCCAGTGAATTCGCACAGAATCAATGGCTTC	CAGCTCGAGCTCGATGGATCCCGTGTTTGTTGGTTTGGC	
VcbHLH1-2	TTCCCTTACCCATCTTCCT	CTGTGTTTGTTGGTTTGGC	
VcbHLH1-2-AD	GCCATGGAGGCCAGTGAATTC TTCCCTTACCCATCTTCCT	CAGCTCGAGCTCGATGGATCCCTGTGTTTGTTGGTTTGGC	
GAPDH	GCTCCCAGCAAGGATGCCCC	CGGAAGGCCATTCCAGTCAACT	
qVcCHS8	TCGGGTTCTAGTCGTTTGCT	ATGGCTTCTCAACTTCGGGA	
qVcCHS13	CCTCGTTGTTTGCTCCGAAA	AGACGAGTTGGAAGAGTGGG	qRT-PCR
qVcCHS21	CGCATGTGTGACAAATCCCA	ACCATATCCTGCCTAGCGTC	
qVcCHI	TCTTTCCTCCGTCGGTCAAA	ACTCTACGGCGCTAACTTGT	
qVcDFR	GCTTCTTGAACGGGGCTATG	CTTCAATGGCCTCGTCGAAG	

qVcF3H	GTGGACGGAGCTTTTGTTGT	GTGATTGGCTCGTCGAGAAC
qVcANS	ACCTGAGAGCCCTAACAACC	CTGTGATCCATTTGCCCTCG
qVcUFGT	ATTGGTGTGAGAGTGGAGGG	GTCCAACAGCCTTCAAAGCA
Actin	TGACCGAATGAGCAAGGAAATTACT	TACTCAGCTTTGGCAATCCACATC
qMdCHS	GGAGACAACTGGAGAAGGACTGGAA	CGACATTGATACTGGTGTCTTCA
qMdDFR	GATAGGGTTTGAGTTCAAGTA	TCTCCTCAGCAGCCTCAGTTTTCT
qMdANS	GAGAAGTATGCCAATGACCAGG	GGCGGTTGCCTCAATGTAAT
qMdUFGT	CCACCGCCCTTCCAAACACTCT	CACCCTTATGTTACGCGGCATGT
